# Functional Characteristics of General Odorant Binding Proteins in the Sugarcane Borer (*Tryporyza intacta*)

**DOI:** 10.3390/genes17091051

**Published:** 2026-08-30

**Authors:** Siyu Liu, Yuwei Hu, Yueyi Li, Piao Luo, Yuying Liu, Jixia Huang, Hui Ai

**Affiliations:** 1 Hubei Key Laboratory of Genetic Regulation and Integrative Biology, Key Laboratory of Pesticide & Chemical Biology of Ministry of Education, School of Life Sciences, Central China Normal University, Wuhan 430079, China; 2 Guangzhou National Agricultural Science and Technology Innovation Center, Guangzhou 510520, China; 3 Academy of Frontier Interdisciplinary Research, Central China Normal University, Wuhan 430079, China; 4 Research Center for Territorial Spatial Conservation, Utilization and Computational Governance, Central China Normal University, Wuhan 430079, China

**Keywords:** *Tryporyza intacta*, general odorant binding protein, protein expression, binding affinities, molecular docking

## Abstract

Background/Objectives: *Tryporyza intacta* is a stem-boring pest that readily causes dead hearts in sugarcane seedlings and dead tops in mature plants, leading to large-scale yield reduction. Reverse chemical ecology studies of this pest contribute to its field biological control. During olfactory recognition, odor molecules are integrated by the olfactory nervous system after interacting with odorant-binding proteins (OBPs) and chemosensory proteins (CSPs), thereby regulating insect behavior. Methods: Two novel full-length general odorant-binding protein (GOBP) genes were cloned from antennal tissues using reverse transcription PCR. Protein sequence analysis was conducted to determine sequence similarity and conserved structural features. The recombinant GOBP1-2 protein was expressed in *Escherichia coli* and purified via Ni-ion affinity chromatography. Fluorescence binding assays were performed to evaluate the binding affinities of GOBP1-2 with various volatile odorant molecules. Results: Protein sequence analysis revealed that the two identified GOBPs shared high sequence similarity with other insect GOBPs and contained the characteristic six-cysteine motif. Fluorescence binding assays demonstrated that GOBP1-2 proteins exhibited differential binding affinities to distinct volatile odorant molecules, indicating selective ligand recognition. Conclusions: These findings suggest that GOBPs and effective volatile odorants likely play a functional role in the olfactory behavioral responses of this moth. This research provides valuable insights for developing field attractants targeting this sugar borer.

## 1. Introduction

Olfactory chemoreception represents a critical survival mechanism in insects, enabling the precise detection of key environmental cues such as food sources, mating partners, and oviposition sites [1]. The evolutionary refinement of the insect olfactory system is exemplified by its remarkable capacity to discriminate among thousands of volatile organic compounds, including host plant volatiles and conspecific pheromones, thereby facilitating ecological adaptation to dynamic environments [2]. The olfactory capacity of insects relies on the recognition of odorant ligands, a process primarily coordinated by odorant-binding proteins (OBPs) [3]. OBPs are small, water-soluble globular proteins that form complexes with odor molecules, facilitating their transport through the antennal lymph to odorant receptors (ORs) located on the dendritic membranes of olfactory sensory neurons. This interaction converts chemical signals into electrical impulses, which are subsequently transmitted to the central nervous system, ultimately guiding appropriate behavioral responses [4].

Functional studies across insect species have highlighted the critical role of OBPs in volatile recognition. For instance, OBP3 and OBP4 of *Chilo auricilius* exhibit strong binding affinities for α-pinene, suggesting their involvement in host volatile detection [5]. Similarly, the *Cnaphalocrocis medinalis* OBP13 protein binds significantly to 23 volatile compounds, with particularly high affinity for six rice volatiles: hexanal, 2-heptanone, nerolidol, 2-tridecanone, methyl salicylate and (E,E)-α-farnesene [6]. In *Plutella xylostella*, PxylOBP2 is implicated in mate-seeking behavior and exhibits synergistic interactions with 11 host plant volatiles, including linalool, nonyl alcohol, α-pinene and β-ionone [7]. In *Apis cerana*, the *OBP14* gene is highly expressed in antennae and displays the strongest binding affinity toward β-ocimene, α-farnesene, eugenol and citronellol, underscoring its essential function in plant odor recognition [8]. Given their structural and functional diversity, OBPs represent promising molecular targets for developing environmentally sustainable pest management strategies [9].

*T. intacta* is a primary pest of sugarcane, causing significant reductions in yield, sugar content, and crop quality, thereby posing a critical threat to sugarcane productivity and farmer income [10,11]. Given the severe damage and frequent outbreaks of this pest, an integrated management strategy has been implemented, primarily relying on chemical control for rapid population suppression, supplemented by physical light traps and cultural practices, to achieve sustained and effective control of *T. intacta* [12,13,14]. However, prolonged reliance on chemical control presents ongoing challenges, including pesticide residues, environmental pollution and unintended harm to natural enemies, all of which adversely affect ecosystems and biodiversity [15]. In contrast, biological control utilizing natural enemies or pheromones offers an environmentally friendly and operationally simple alternative that is not constrained by factors such as plant height or geographical conditions. This approach effectively maintains ecological balance and holds considerable promise for broader application [16]. Therefore, further investigation into the General odorant-binding proteins (GOBPs) protein of *T. intacta* may provide critical insights for the development of attractant-based trapping strategies [17].

General odorant-binding proteins were present in many Lepidoptera insects and served as an important class of broad-spectrum odorant-binding proteins. Given the important biological functions of OBPs in insect chemosensory physiology, this study aimed to investigate the role of GOBP1 and GOBP2 in *T. intacta* in recognition of host plant volatiles. The full-length cDNA sequences of *GOBP1* and *GOBP2* (Table S1) derived from transcriptome data were cloned, and the recombinant proteins were heterologously expressed in *Escherichia coli* to enable functional characterization. The binding affinities of these two general odorant-binding proteins to volatile compounds derived from sugarcane were subsequently evaluated using fluorescence competitive binding assays. Accordingly, this study aimed to elucidate the ligand-binding properties of GOBP1 and GOBP2, thereby establishing a molecular basis for understanding host plant recognition in *T. intacta* and facilitating the development of behavior-based pest management strategies.

## 2. Materials and Methods

### 2.1. Multisequence Alignment and Phylogenetic Analyses of GOBPs

Amino acid sequences of GOBPs from *T. intacta* and other lepidopteran species (Table S1) were aligned using DNAMAN (v6.0) software. A neighbor-joining phylogenetic tree was constructed using MEGA11 software based on the distance method, supported by 1000 bootstrap replicates. The GOBP1 and GOBP2 protein sequences from 36 Lepidoptera insects were collected, and signal peptides were identified by the SignalP server and removed prior to analysis. Conserved motif analysis was performed using the MEME suite (version 5.5.5).

### 2.2. Homologous Sequence Motif and Conservation Analysis of GOBP1-2 Proteins

The protein sequences of GOBP1-2 without signal peptides were integrated with homologous sequences into a single text document and subsequently renamed in FASTA format. Conserved motif prediction was performed using the MEME online platform with appropriately configured parameters for the signal peptide-removed GOBP1-2 protein sequences and their homologs. The MEME HTML output was selected for result visualization. Based on the analysis results, redundant amino acids at the termini of each sequence were trimmed, and DNAMAN was used to ensure uniform sequence lengths across all entries. The processed sequences were consolidated into a FASTA-formatted document, and conserved sequence features were visualized using the WEBLOGO online platform to generate sequence logos. To obtain standardized motif analysis figures, the motif analysis results were downloaded from the MEME website in XML format and further refined using TBtools (v2) software to optimize the graphical output, thereby enabling in-depth analysis of specific binding sites in GOBP1-2 proteins and their homologous sequences from other species (Table S1).

### 2.3. Prokaryotic Expression and Purification of TintGOBPs

The purified fragments were ligated into the linearized expression vector pET32a(+) using T4 DNA ligase at 22 °C for 20 min. The ligation mixture was then transformed into *Escherichia coli* BL21 (DE3) competent cells (TransGen Biotech, Beijing, China), and recombinant protein expression was induced with isopropyl β-D-1-thiogalactopyranoside (IPTG) at a final concentration of 0.5 mM. Sodium dodecyl sulfate polyacrylamide gel electrophoresis (SDS-PAGE) analysis revealed that the expressed TintGOBPs were present in inclusion bodies. Following established protocols, the recombinant proteins were subjected to denaturation and renaturation, and the soluble fractions were subsequently purified and concentrated using Ni-NTA His Bind Resin.

### 2.4. Fluorescence Binding Assays

The fluorescent probe N-phenyl-1-naphthylamine (1-NPN) and the test ligands were dissolved in methanol to prepare 1 mM stock solutions. Binding assays were conducted in 2 mL of 20 mM Tris-HCl buffer (pH 7.4) containing 2 µM TintGOBP protein and 2 µM 1-NPN. To determine the binding affinity of 1-NPN to each TintGOBP protein, aliquots of the 1 mM 1-NPN stock solution were incrementally added to a 2 µM protein solution to achieve final concentrations ranging from 0 to 96µM. Three biological replicates were performed for the 1-NPN binding curve with TintGOBP protein. Fluorescence intensity measurements were performed at ambient temperature using an F-4700 fluorescence spectrophotometer (Hitachi, Tokyo, Japan). Nonlinear regression analysis was conducted with GraphPad Prism 8 software to calculate the dissociation constant (Kd) of 1-NPN binding to TintGOBPs. For competitive binding assays, 2 mL of the solution containing TintGOBP protein and 1-NPN was titrated with each test ligand to final concentrations ranging from 2 to 48 µM. Fluorescence intensity was recorded after a 2 min incubation using the same spectrophotometer. The inhibitory constant (Ki) for each ligand was calculated using the equation Ki = IC_50_/(1 + [1-NPN]/K1-NPN), where IC_50_ represents the concentration of the competitor required to displace half of the initial fluorescence intensity of the TintGOBP/1-NPN complex, K1-NPN is the dissociation constant of the TintGOBP/1-NPN complex, and [1-NPN] is the free concentration of 1-NPN. All dissociation constants (Kd) and inhibitory constants (Ki) are reported as mean ± standard error.

### 2.5. Protein Modeling and Molecular Docking

Suitable template structures exhibiting sequence or functional similarity to TintGOBP1 and TintGOBP2 were identified from the Protein Data Bank (PDB) and other protein structure databases. Three-dimensional models of TintGOBP1 and TintGOBP2 were generated using molecular modeling software (SWISS-MODEL, http://swissmodel.expasy.org/) based on the selected templates. The amino acid sequences of TintGOBP1 and TintGOBP2 were aligned with the template sequences to ensure accurate residue correspondence, and energy minimization and optimization procedures were applied to refine the geometric and energetic properties of the resulting models. Model quality was assessed using validation tools such as PROCHECK (v3.0) and WHATCHECK (v8.0) to evaluate stereochemical quality and overall reliability. The final structural models were visualized and interpreted using molecular graphics software PyMOL (v3.0). The three-dimensional structures of TintGOBP1, TintGOBP2 and the target ligands were obtained, followed by structural preparation involving removal of water molecules and optimization of ligand conformations to ensure suitability for docking. A grid box was defined around the putative active site of each protein to evaluate ligand binding fitness and interaction potential. Molecular docking was performed using AutoDock to predict the optimal binding poses of the ligands with the protein structures, thereby improving the reliability of the predicted binding modes. Docking results were analyzed based on docking scores, binding affinities, and potential intermolecular interactions, including hydrogen bonds and hydrophobic contacts. The predicted binding modes and interactions were further validated using experimental data or complementary computational approaches.

## 3. Results

### 3.1. Amino Acid Sequence Analysis of TintGOBP1-2 and Alignment to Homologs of Other Species

**Figure 1:**
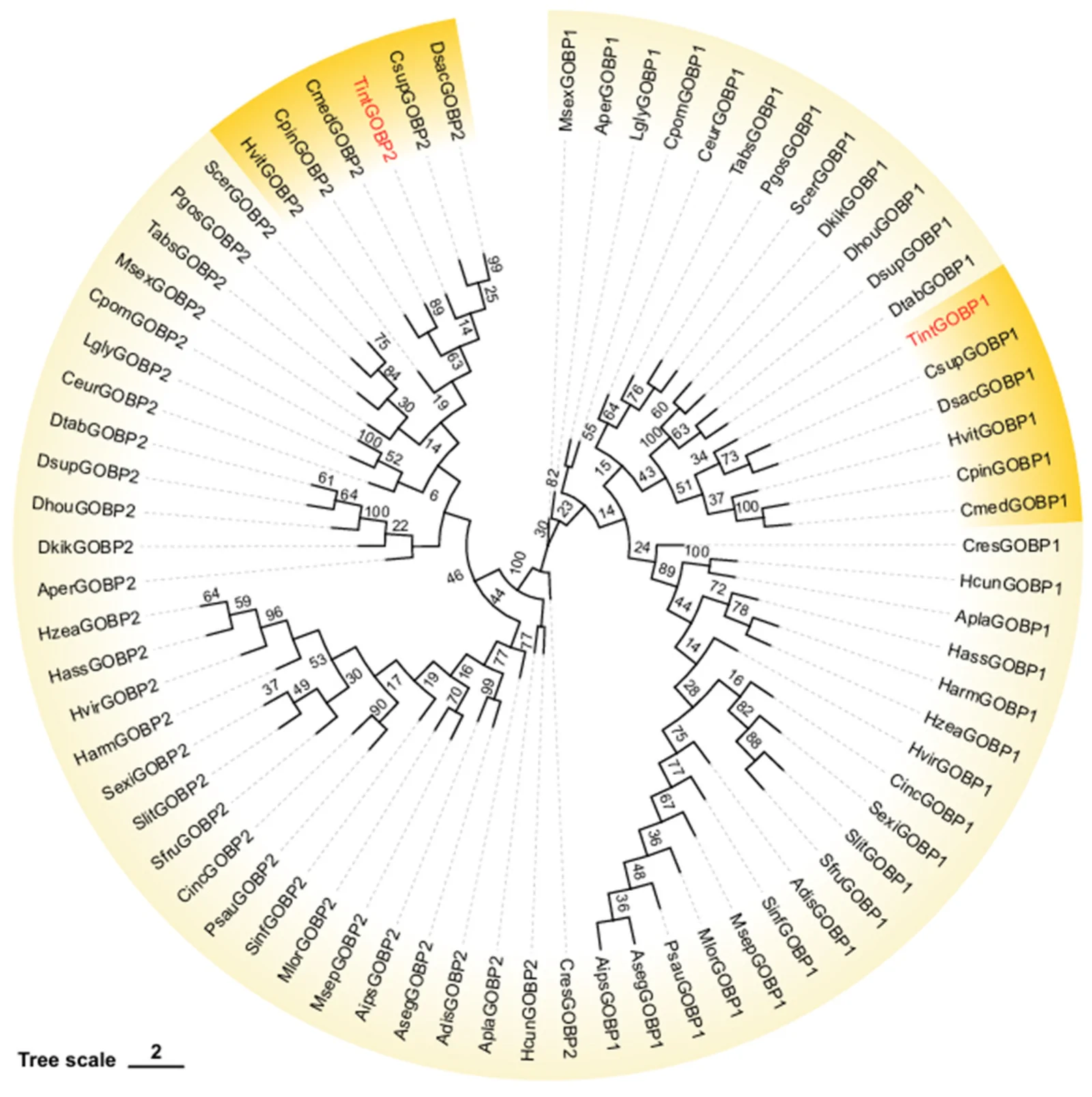
Phylogenetic analysis of general odorant binding proteins (GOBPs) from *T. intacta* and other moths.

To investigate the evolutionary relationships of TintGOBP1 and TintGOBP2 within the Lepidoptera, a phylogenetic tree was constructed using the maximum likelihood method implemented in MEGA X, based on GOBP protein sequences from Lepidoptera species with high homology identified through BLAST analysis. The resulting tree exhibited distinct clustering patterns. TintGOBP1 clustered with HvitGOBP1, CmedGOBP1, CpinGOBP1, DsacGOBP1 and CsupGOBP1 to form a well-supported clade. Similarly, TintGOBP2 grouped with HvitGOBP2, CmedGOBP2, CpinGOBP2, DsacGOBP2 and CsupGOBP2 (Figure 1). This phylogenetic topology not only provides strong evidence for the distinct subtype differentiation between GOBP1 and GOBP2 across Lepidoptera species, but also reveals a high degree of evolutionary conservation among these homologous proteins, suggesting their close phylogenetic relationships and potential functional constraints during evolution.

**Figure 2:**
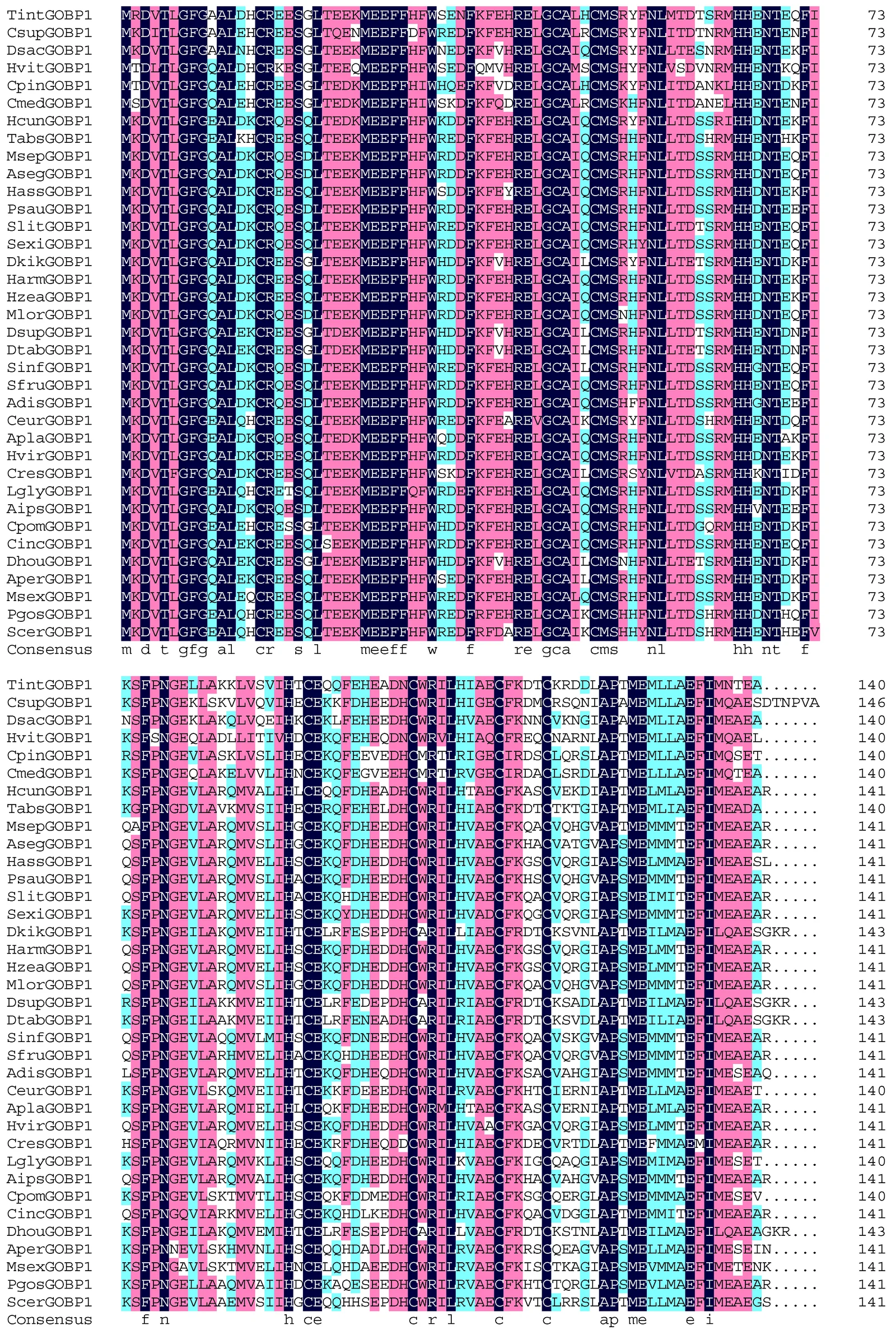

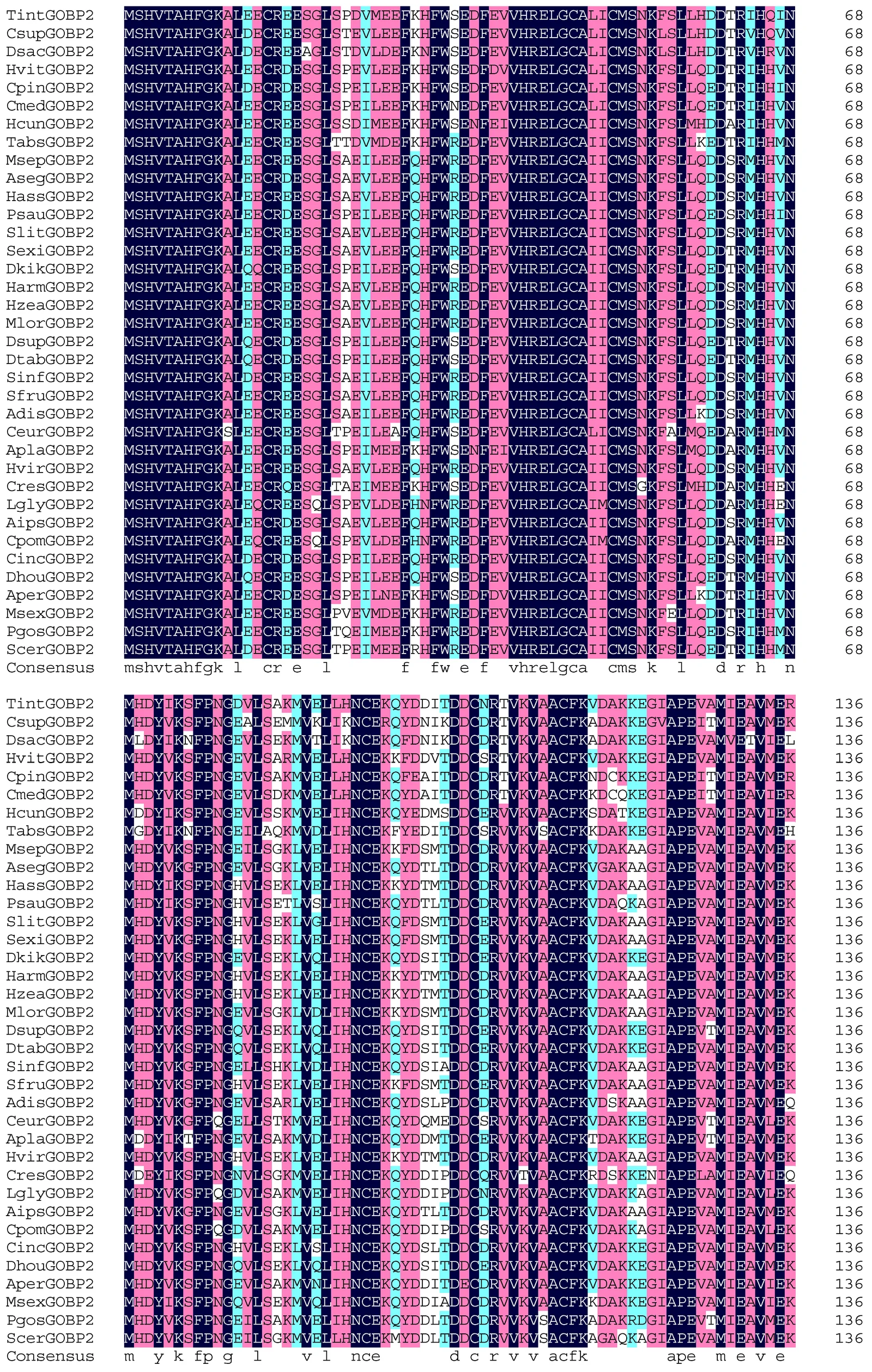
Alignment of GOBPs from 36 species of Lepidoptera.

TintGOBP1 and TintGOBP2 comprise 140 and 149 amino acid residues, respectively, and are classified as canonical members of the general odorant-binding protein (GOBP) family. Comprehensive sequence analysis revealed that both proteins possess a conserved structural motif characterized by six cysteine residues arranged in a specific spatial configuration following the pattern C1-X15-39-C2-X3-C3-X21-44-C4-X7-12-C5-X8-C6. This distinctive cysteine framework not only provides strong evidence for the evolutionary conservation of GOBPs across insect taxa, but also suggests significant functional constraints acting on these proteins (Figure 2). The presence of these highly conserved domains is considered essential for maintaining structural integrity and functional specificity, particularly in ligand binding and olfactory signal transduction. Multiple-sequence alignment and phylogenetic analysis of TintGOBP1 and TintGOBP2, together with their orthologs from other Lepidoptera species, were conducted using DNAMAN software. The results demonstrated a high degree of sequence homology among GOBP amino acid sequences across diverse taxa, offering compelling molecular evidence for the evolutionary conservation of GOBPs within the order Lepidoptera (Table S1).

### 3.2. Motif Pattern Analysis

Conserved motif analysis conducted using the MEME software suite revealed that TintGOBP1-2 and other Lepidoptera GOBPs contain four conserved structural motifs, designated Motif 1 through Motif 4, within their GOBP1 sequences. Among these, Motif 1, Motif 2 and Motif 4 exhibited a compact and uniform spatial distribution, suggesting their potential functional significance in maintaining structural integrity and ligand-binding properties. Structural analysis identified a conserved pheromone-binding protein (PhBP) domain spanning residues 11–116, characterized by six strategically positioned cysteine residues located between Motif 1 and Motif 4. Among the identified motifs, Motif 1 and Motif 2 represent the most extensive structural domains, each comprising 50 amino acid residues. Motif 1 contains three conserved cysteine residues, whereas Motif 2 harbors two. In contrast, Motif 4 constitutes the shortest structural element and lacks cysteine residues, while Motif 3 contains an additional conserved cysteine residue. These motifs are ubiquitously present in GOBP proteins and exhibit a tightly packed spatial arrangement, following a consistent sequential order of “Motif 2–Motif 1–Motif 4–Motif 3.” The minimal E-values associated with each motif further corroborate their robustness and underscore their functional importance in the structural organization and ligand-binding properties of these proteins.

**Figure 3:**
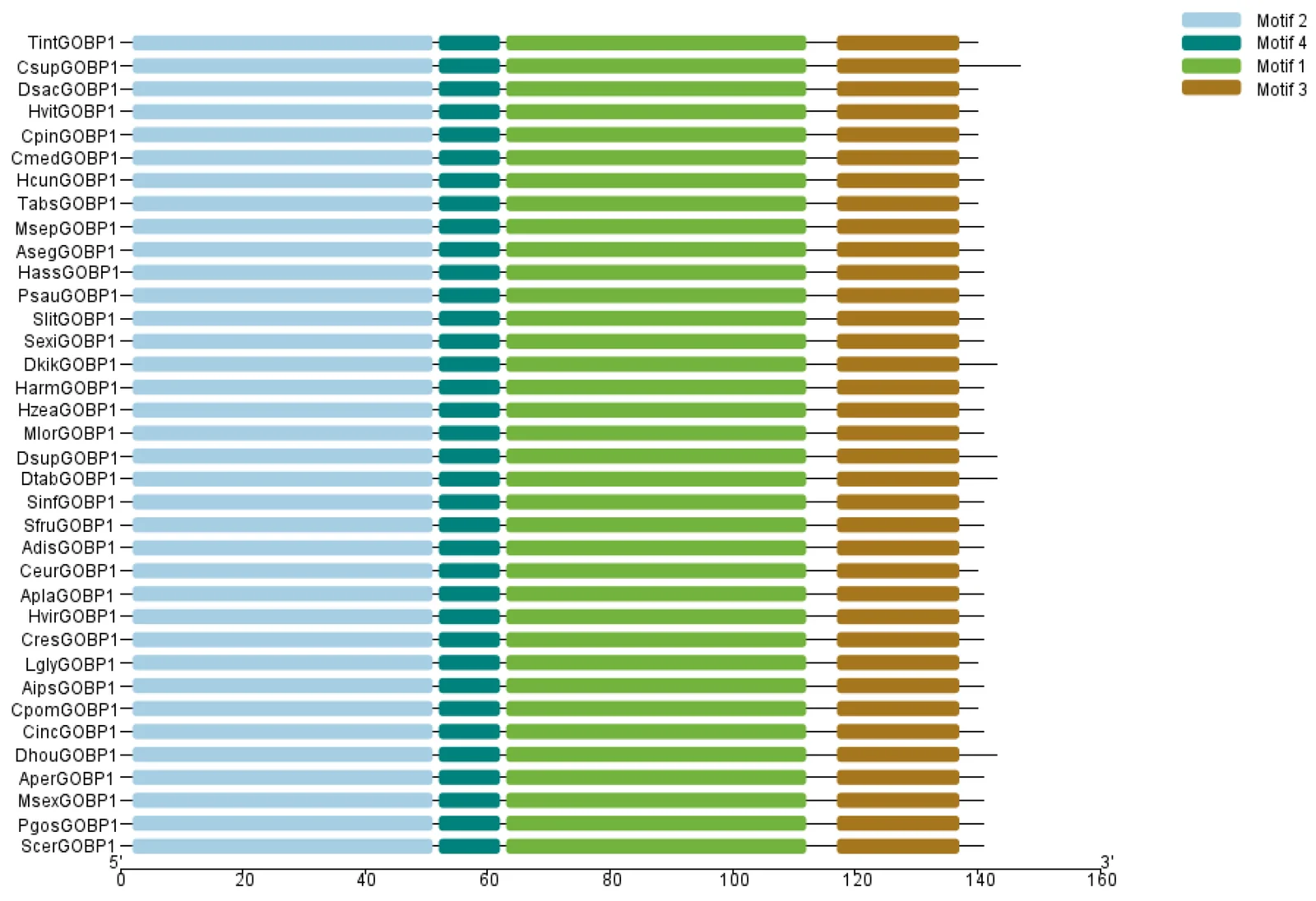
Motif analysis was performed among 36 GOBP1 proteins from Lepidoptera.

**Figure 4:**
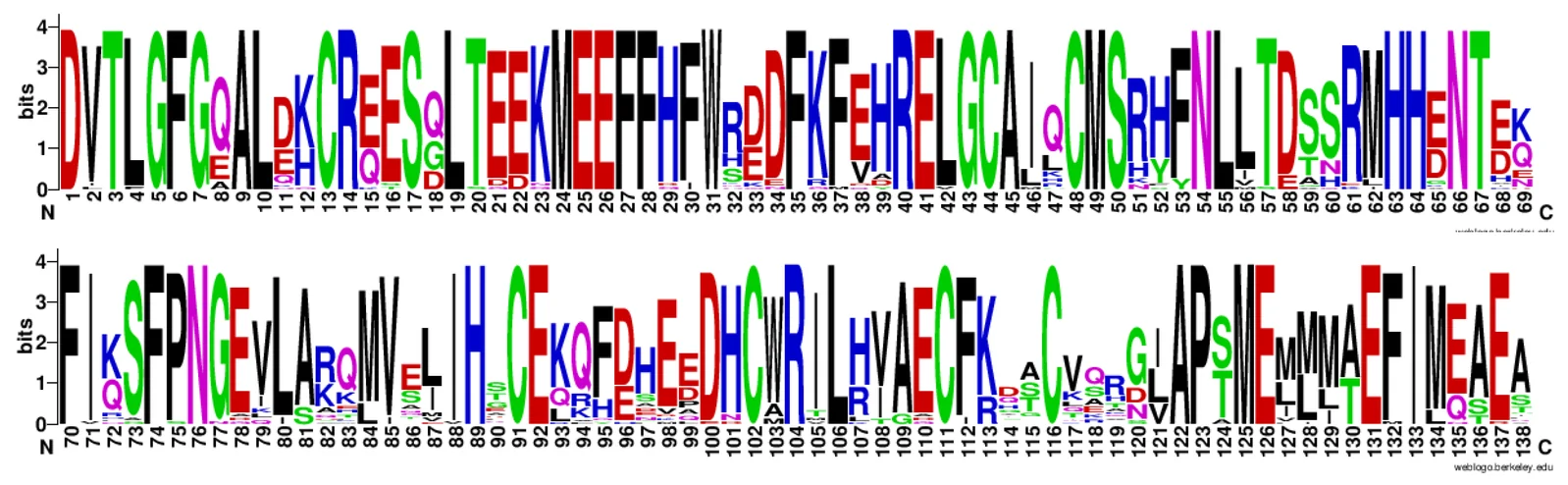
Logo of 36 GOBP1 protein sequences from 36 Lepidoptera species.

Examination of the GOBP1 protein sequences from 36 Lepidoptera species (Figure 3 and Figure 4) revealed a distinct pattern characterized by high information content across multiple amino acid residues, indicating substantial conservation within the motif regions. This pronounced sequence similarity within the structural domains further supports the high degree of evolutionary conservation among GOBP1 homologs.

**Figure 5:**
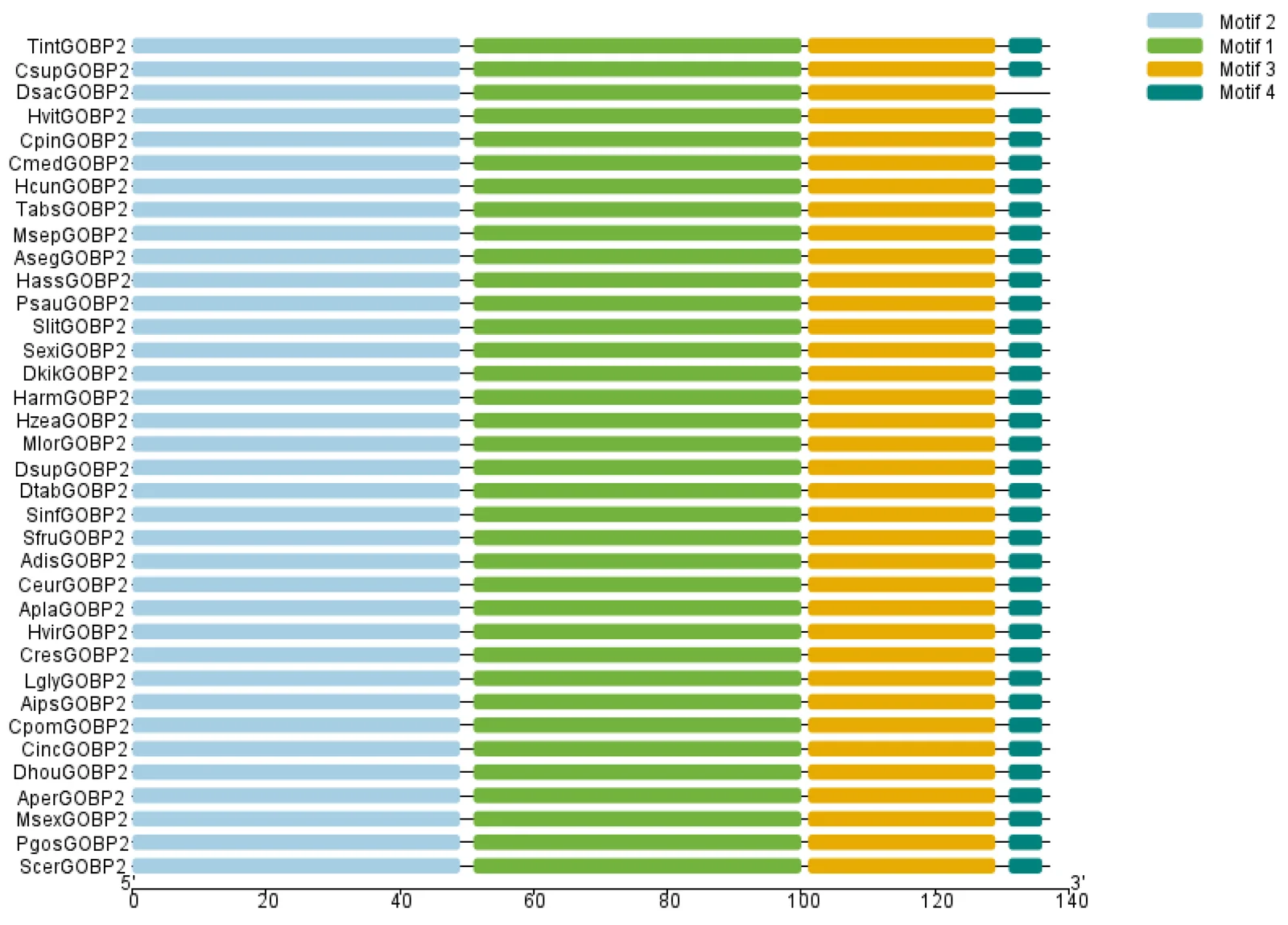
Motif analysis was performed among Lepidoptera 36 GOBP2 proteins.

Motif analysis of TintGOBP2 homologous sequences in *T. intacta* revealed a high degree of conservation in motif architecture among GOBP2 proteins. Four distinct motifs, designated Motif 1 through Motif 4, were identified, reflecting their evolutionary conservation. Notably, DsacGOBP2 exhibited a “Motif 2–Motif 1–Motif 3” arrangement, diverging from the “Motif 2–Motif 1–Motif 3–Motif 4” pattern observed in the remaining GOBP2 proteins. The absence of Motif 4 in DsacGOBP2 suggests potential functional divergence or specialized adaptation of this protein. All other sequences displayed a tightly organized and consistent motif structure, with a region spanning residues 11–115 encompassing the pheromone-binding protein (PhBP) domain (Figure 5). Additionally, six conserved cysteine (Cys, C) residues were identified within these GOBP2 proteins, a hallmark feature of odorant-binding proteins that is critical for their structural integrity and ligand-binding capabilities. These findings are consistent with results from multiple-sequence alignment and motif analyses, collectively underscoring the pronounced structural and functional conservation among these proteins.

**Figure 6:**
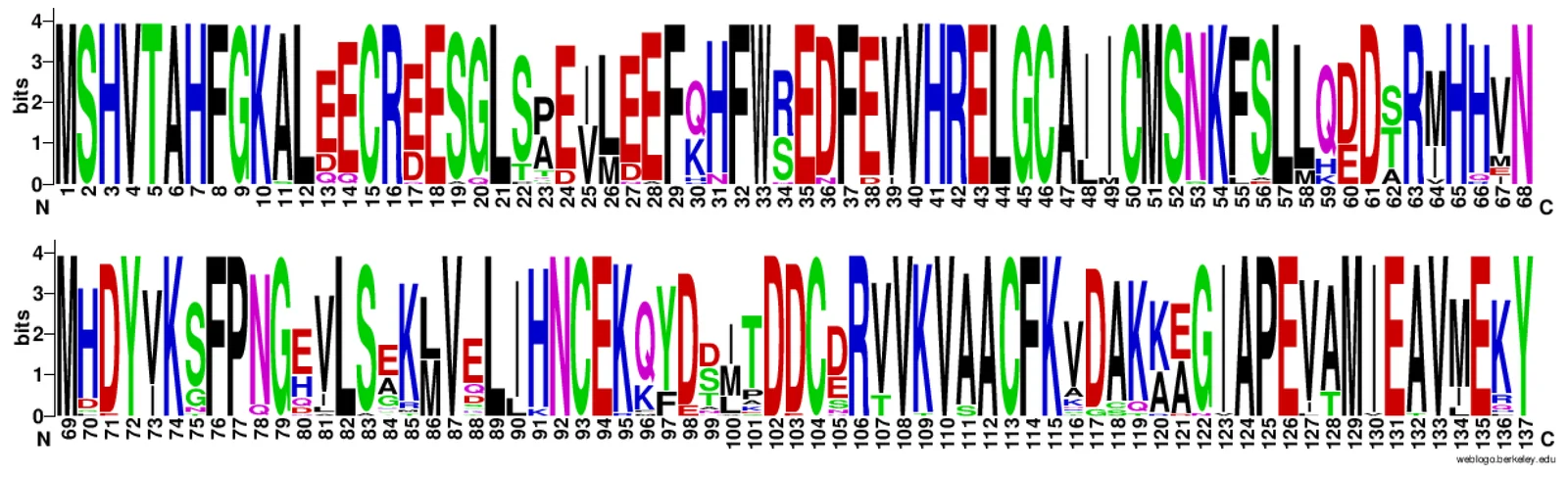
Motif analysis was performed among 36 Lepidoptera GOBP2 proteins.

In the GOBP2 protein sequences of 36 species, a notable regularity is observed, with many amino acid fragments reaching their maximum quantity, indicating significant similarity among the structural domain sequences. Consequently, it suggests a high level of conservation in the motifs within this sequence (Figure 6).

### 3.3. Expression and Purification of TintGOBP1-2

**Figure 7:**
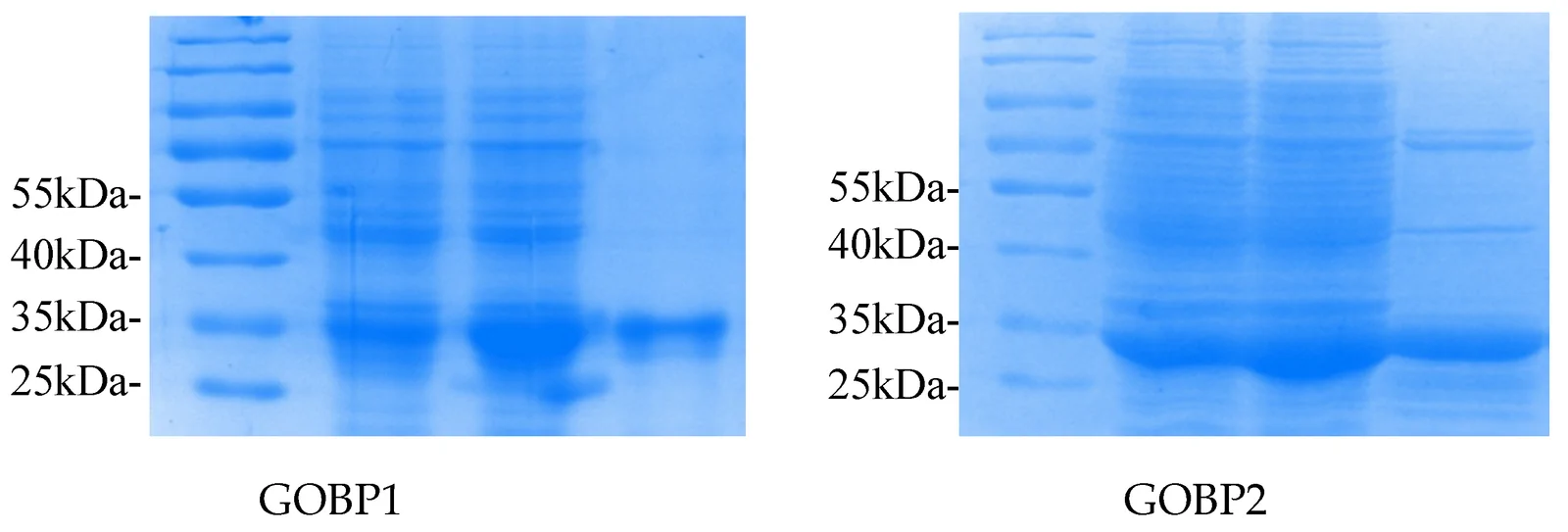
Expression and purification of TintGOBP1-2 proteins. Lane 1: before induction with IPTG; Lane 2: supernatant of the solubilized protein; Lane3: target proteins.

The recombinant plasmid pET-32a(+)/TintGOBPs was transformed into competent *Escherichia coli* BL21 (DE3) cells for heterologous expression of the target proteins. Following induction with IPTG, the recombinant proteins were expressed in soluble form, with distinct protein bands observed between 25 and 35 kDa (Figure 7), corresponding to the predicted molecular weights of TintGOBP1 and TintGOBP2.

### 3.4. Fluorescent Ligand Binding Activities

To elucidate the molecular mechanisms underlying host plant volatile recognition in *T. intacta*, fluorescence competitive binding assays were conducted to evaluate the binding properties of its general odorant-binding proteins (GOBPs) to 22 plant volatiles. The recombinant GOBPs were expressed in a prokaryotic system, predominantly recovered in the soluble fraction, and purified by affinity chromatography for subsequent binding studies. Using 1-NPN as a fluorescent probe, competitive binding assays were performed with key sugarcane leaf volatiles, including linalool, cis-3-hexen-1-ol and acetophenone. Scatchard analysis revealed a linear interaction between TintGOBP1/2 and 1-NPN, indicative of a single, saturable binding site. Fluorescence intensity of the TintGOBP and 1-NPN complex decreased progressively with increasing concentrations of competitor odorants, confirming 1-NPN as a suitable probe for investigating GOBP–plant volatile interactions. These findings provide insights into the binding specificity and functional roles of GOBPs in host recognition.

**Figure 8:**
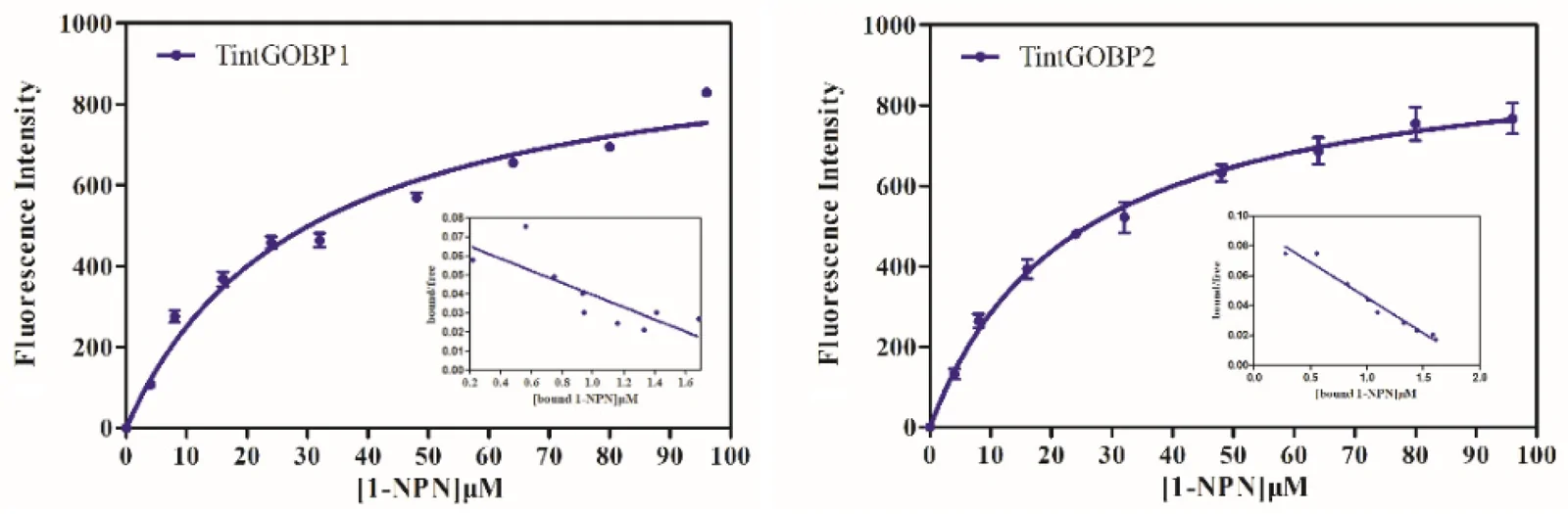

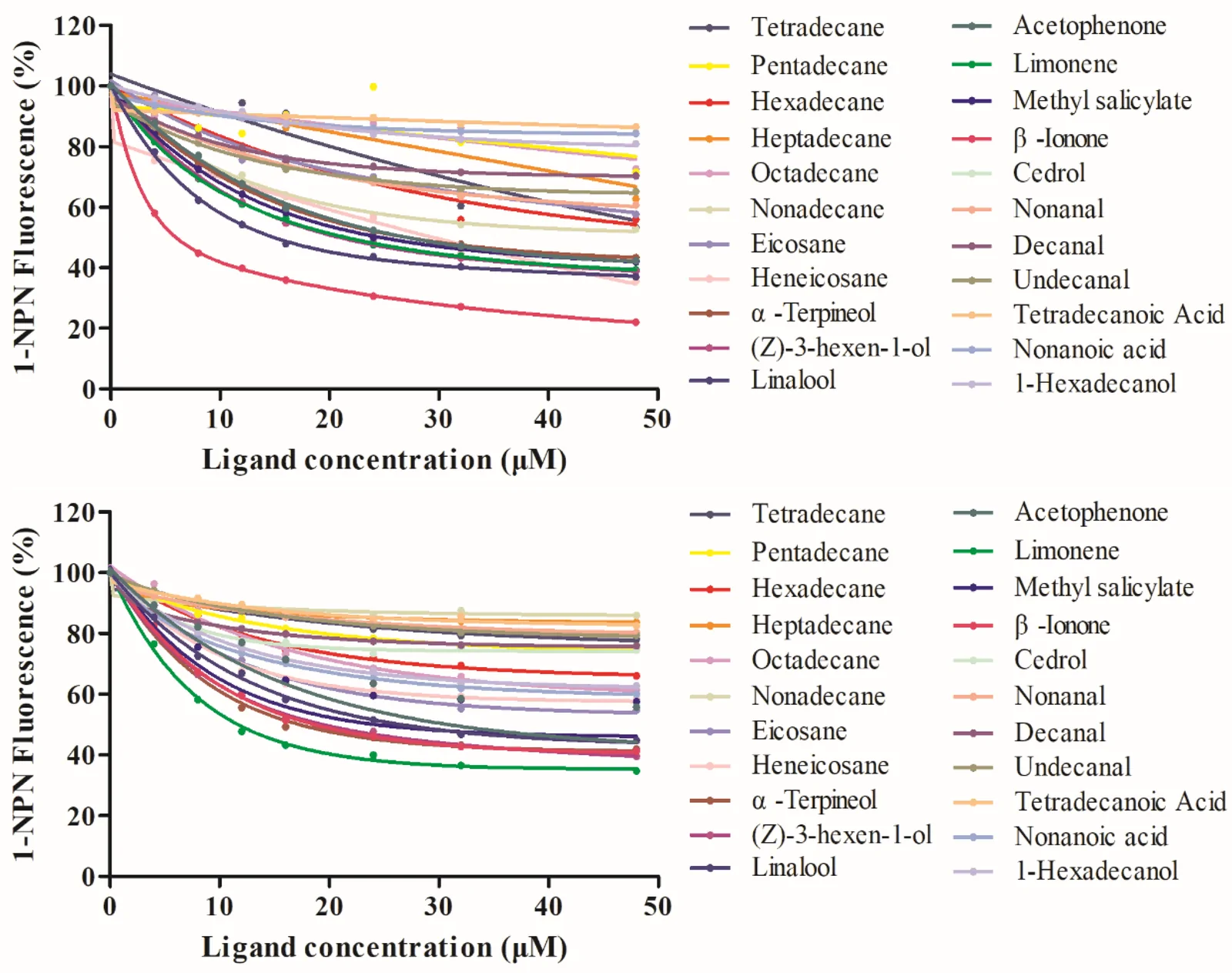
Binding curves of 1-NPN and competitive binding activities of volatile components to TintGOBP1-2 proteins.

The binding affinities of TintGOBP1 and TintGOBP2 for 22 host plant volatiles were determined (Figure 8), with corresponding IC50 values and dissociation constants (Ki) summarized in Table 1. TintGOBP1 exhibited strong binding to linalool, (Z)-3-hexen-1-ol, acetophenone, limonene, methyl salicylate, terpineol, β-ionone and heneicosane, showing the highest affinities for linalool (Ki = 16.31 μM) and β-ionone (Ki = 10.28 μM). Similarly, TintGOBP2 displayed strong binding to linalool, (Z)-3-hexen-1-ol, limonene, terpineol and β-ionone, with the strongest affinities observed for limonene (Ki = 13.31 μM) and terpineol (Ki = 16.70 μM). Collectively, these results highlight the distinct ligand-binding profiles of TintGOBP1 and TintGOBP2.

### 3.5. Molecular Docking

**Figure 9:**
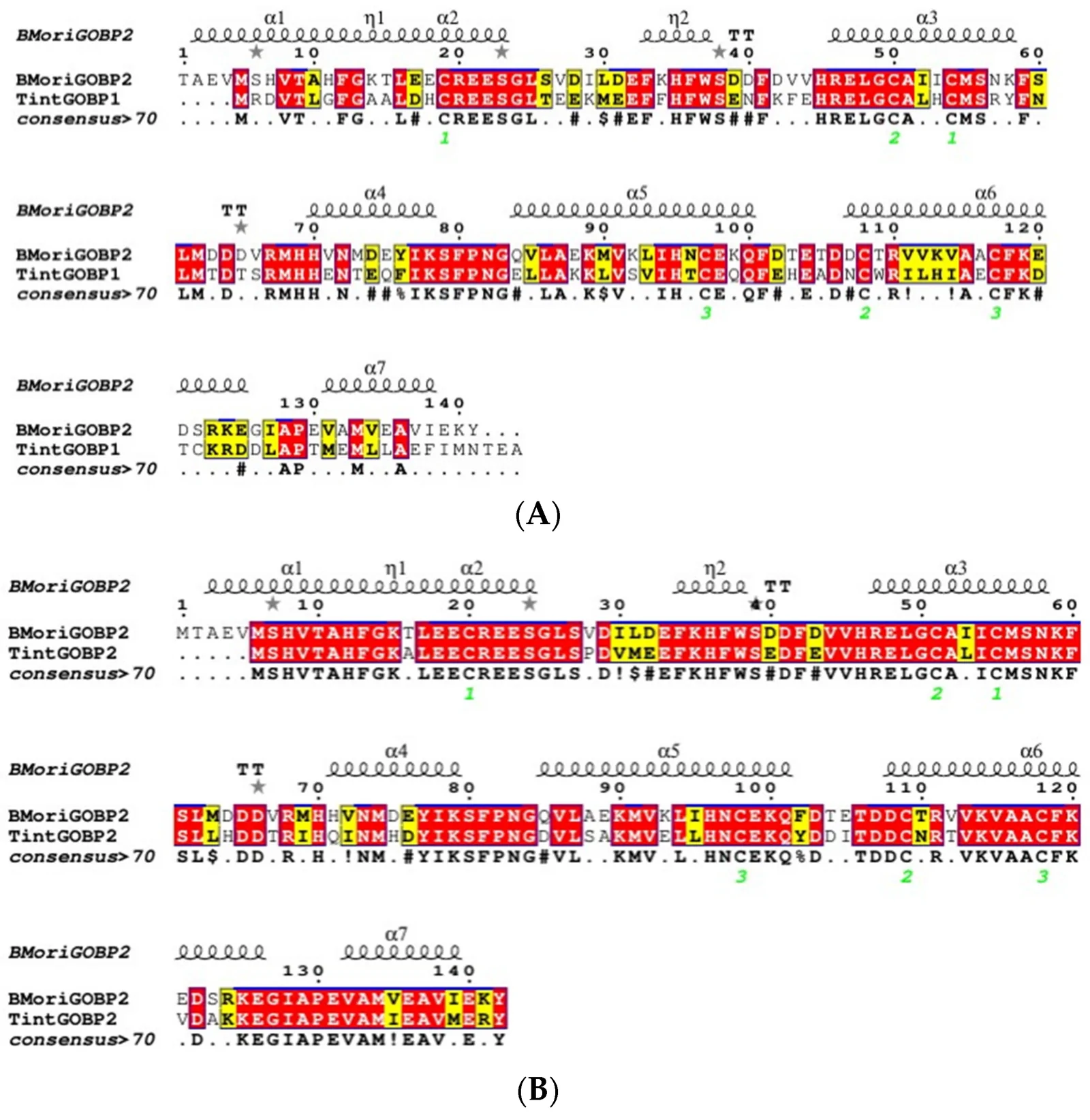
Multiple-sequence alignment of TintGOBP1 (**A**) and TintGOBP2 (**B**) with template protein BmoriGOBP2.

**Figure 10:**
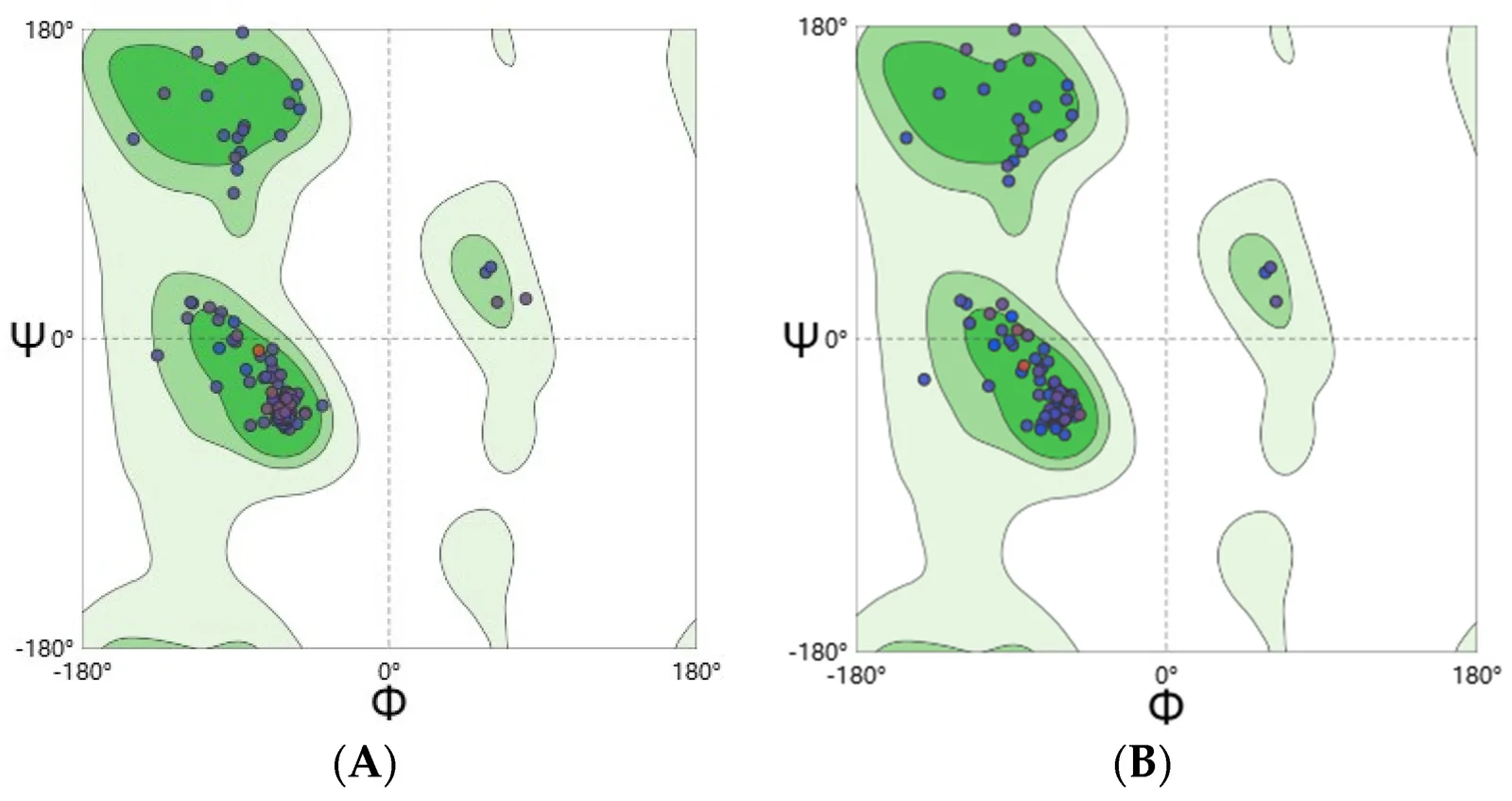
Evaluation of TintGOBP1-2 proteins’ conformational rationality.

To further investigate the interactions between active odorants and TintGOBP1 and TintGOBP2, *Bombyx mori* GOBP2 (BmoriGOBP2, 2wc6) was selected as a template for homology modeling. Sequence alignment revealed high similarity between TintGOBP1 and TintGOBP2 with BmoriGOBP2, showing sequence identities of 47.22% and 73.94%, respectively (Figure 9). Evaluation of the modeled protein structures indicated that the majority of amino acid residues were located within the allowed and generously allowed regions of the Ramachandran plot, confirming that the models conform to stereochemical constraints. Therefore, the template protein was considered suitable for subsequent molecular docking studies (Figure 10).

**Figure 11:**
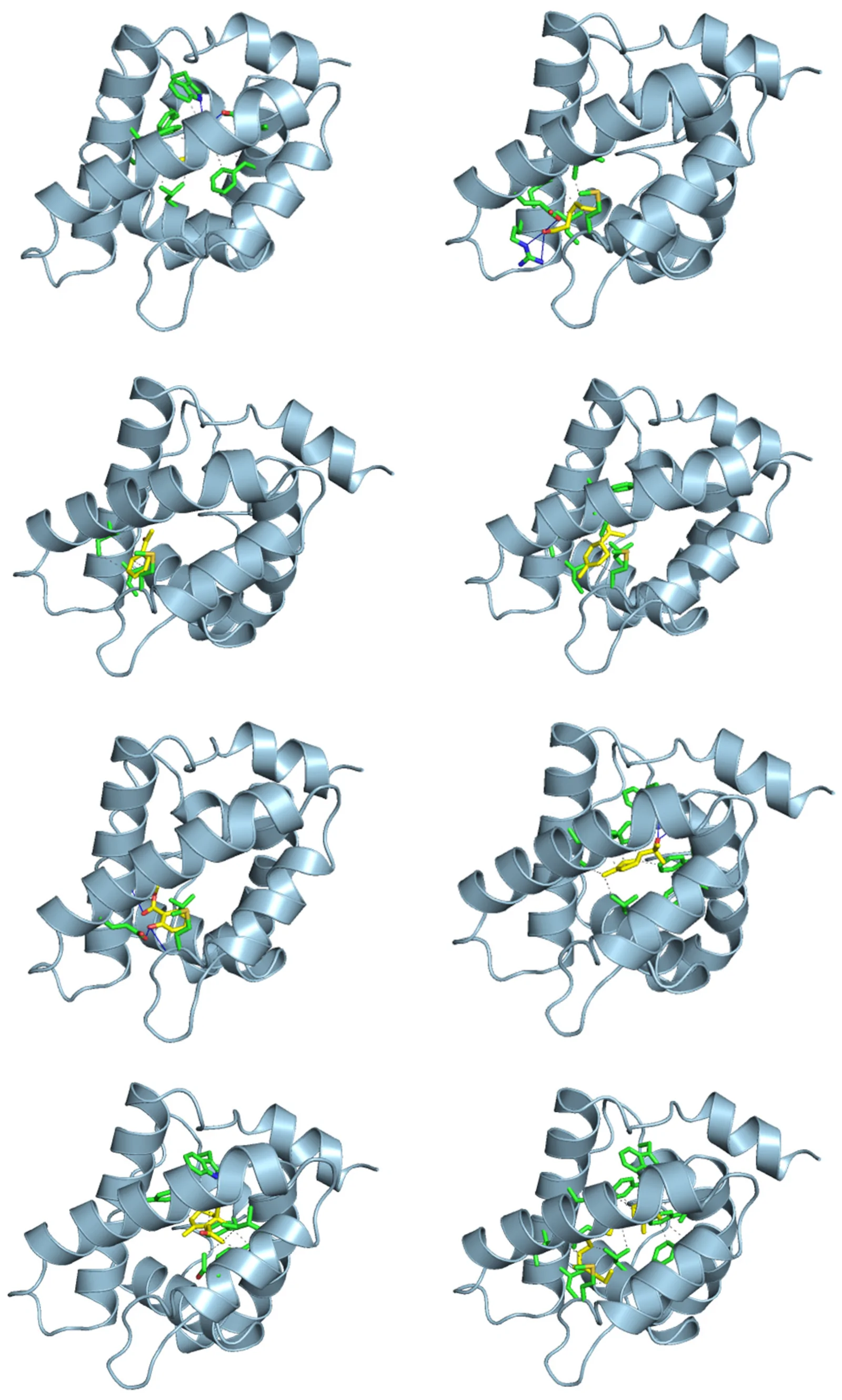
Molecular docking results of TintGOBP1 with ligands (Linalool, (Z)-3-Hexen-1-ol, Acetophenone, Limonene, Methyl salicylate, Terpineol, β-Ionone, Heneicosane).

**Figure 12:**
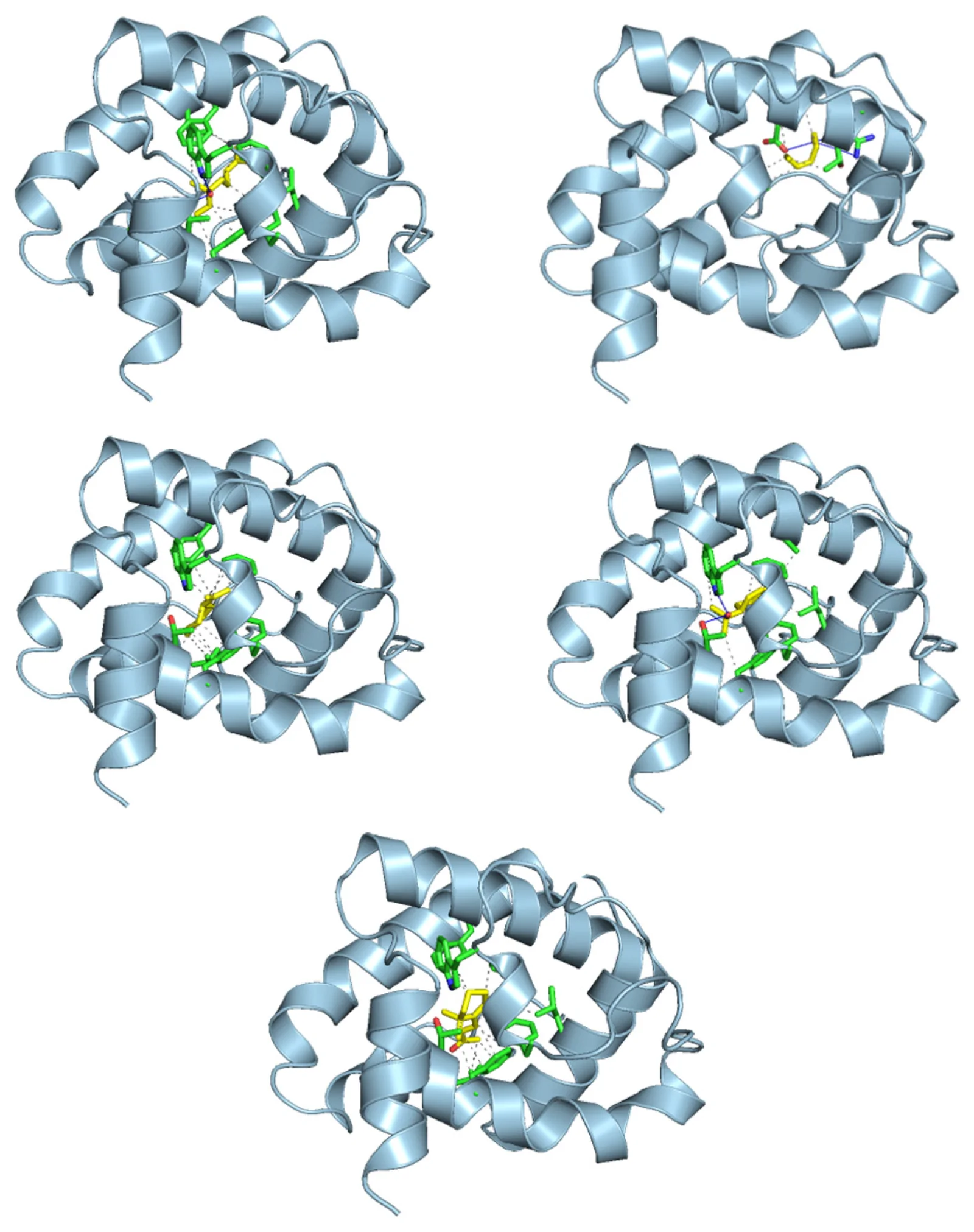
Molecular docking results of TintGOBP1 with ligands (Linalool, (Z)-3-Hexen-1-ol, Limonene, Terpineol, β-Ionone).

Using SWISS-MODEL, three-dimensional structures of GOBP1-2 proteins were constructed via homology modeling. Structural analysis identified seven α-helices and three conserved disulfide bonds, which stabilize the protein framework. Based on fluorescence competitive binding assays, host plant volatiles with high binding affinity were selected for molecular docking with the 3D protein models to explore interaction mechanisms and binding sites between the sugarcane borer’s olfactory proteins and host plant pheromones. The docking results were visualized using PyMOL, generating a 3D model of TintGOBP1-2 bound to high-affinity host plant pheromone ligands (Figure 11 and Figure 12). Statistical analysis of ligand interaction sites within the binding cavity of TintGOBP1-2 (Table 2) revealed that hydrophobic interactions and hydrogen bonds mediate ligand binding. Free binding energy analysis demonstrated strong affinity between GOBP1-2 and ligands such as Linalool, (Z)-3-Hexen-1-ol, Acetophenone, Limonene, Methyl salicylate, Terpineol, β-Ionone and Heneicosane, with predominantly negative binding energies. Hydrogen bonding was observed between GOBP1 and most ligands, with key residues including Thr5, Trp33, and Met64. GOBP2 exhibited strong binding to Linalool, (Z)-3-Hexen-1-ol, Limonene, Terpineol and β-Ionone, with potential key residues Thr5, Trp33, Tyr77 and Gln95, requiring further validation via site-directed mutagenesis.

## 4. Discussion

In recent years, reverse chemical ecology has emerged as a promising strategy for identifying behaviorally active compounds in insects by exploiting the selective binding properties of odorant-binding proteins as molecular targets. *T. intacta* is a major pest in sugarcane production, and its management encompasses biological, chemical, genetic and trapping-based approaches [18]. Biological control leverages natural enemies such as parasitic wasps and predatory insects to suppress pest populations by targeting borer eggs in the field [19]. Regarding trapping strategies, Liu et al. synthesized the sex pheromone of *T. intacta* and demonstrated in field trials that the highest trapping efficacy was achieved with a 70:30 ratio of E-11-hexadecenal to Z-11-hexadecenal [20]. Compared with conventional insecticide application, this approach substantially reduces chemical residues in the field and mitigates environmental impact [12]. Consequently, elucidating the functional roles of odorant-binding proteins in *T. intacta* may provide a theoretical foundation for the development of more effective control strategies against this pest.

OBPs are small, water-soluble extracellular proteins predominantly localized in the sensillar lymph that bathes the dendritic endings of olfactory sensory neurons [21]. These proteins represent a key evolutionary adaptation in terrestrial insects, facilitating the solubilization and transport of hydrophobic odor molecules and reflecting the evolutionary refinement of the olfactory system. Previous studies have classified OBPs based on the number of conserved cysteine residues; canonical OBPs are characterized by six conserved cysteines that form three intramolecular disulfide bridges, which are essential for structural stability [8]. In the present study, TintGOBP1 and TintGOBP2 exhibited significant sequence homology with 36 GOBP orthologs from other Lepidoptera species, all of which retain the complete set of six cysteine residues. This confirms their classification as members of the classical OBP family and underscores the evolutionary conservation of this structural motif. The second and third cysteine residues are separated by three amino acid residues, whereas the fifth and sixth are separated by eight residues, conforming to the conserved pattern C1-X15-39-C2-X3-C3-X21-44-C4-X7-12-C5-X8-C6, suggesting a critical role for these regions in protein stability and function.

The binding characteristics of TintGOBP proteins were further investigated using 22 volatile compounds shared by sugarcane (*Saccharum officinarum*) and maize (Zea mays). In a related study, Duan et al. reported that recombinant CmedOBP13 from the rice pest *Cnaphalocrocis medinalis* exhibited strong binding affinities (Ki < 20 μM) to six rice volatiles: hexanal, 2-heptanone, nerolidol, 2-tridecanone, methyl salicylate and (E,E)-α-farnesene [6]. Similarly, Li et al. demonstrated that GpylGOBP1 and GpylGOBP4 from the mulberry pest *Glyphodes pyloalis* showed specific binding affinities to three and four host plant-derived volatiles, respectively, implicating their involvement in olfactory-mediated host recognition [22]. Collectively, these findings indicate that OBPs possess strong binding capacities and can selectively recognize odor molecules. In the present study, fluorescence competitive binding assays revealed that TintGOBP1 did not bind to six aliphatic ligands, exhibited moderate binding affinity to three ligands, and showed weak affinity to five ligands. Notably, TintGOBP1 displayed the strongest affinity for β-ionone. These volatile components may interact with other OBPs expressed in the olfactory sensilla of *T. intacta* antennae, contributing to host plant attraction and detection. TintGOBP2 exhibited significant binding affinities to five volatile compounds, with particularly strong interactions observed for limonene and terpineol, respectively, suggesting a specialized role for GOBP2 in the recognition and transport of these specific volatiles.

To further elucidate the molecular mechanisms underlying olfactory chemoreception in *T. intacta*, homology modeling and molecular docking were employed to predict interaction sites between olfactory proteins and ligand compounds. Molecular docking analysis revealed distinct ligand-binding profiles for the two GOBP proteins. TintGOBP1 demonstrated significant binding affinities to eight volatile organic compounds: linalool, (Z)-3-hexen-1-ol, acetophenone, limonene, methyl salicylate, terpineol, β-ionone and heneicosane, with key interacting residues including Thr5, Trp33 and Met64. In contrast, TintGOBP2 exhibited specific binding to five ligands: linalool, (Z)-3-hexen-1-ol, limonene, terpineol and β-ionone mediated by conserved residues Thr5, Trp33, Tyr77 and Gln95 via hydrogen bonding and hydrophobic interactions.

In conclusion, the present findings, together with previous studies, suggest that female *T. intacta* may utilize key floral volatiles to locate suitable host plants. The identification of these volatiles and their cognate binding proteins may facilitate the development of artificial attractants, thereby contributing to more effective monitoring and integrated management strategies for this important sugarcane borer in the field.

## Figures and Tables

**Table 1:** The binding constants of different ligands with TintGOBP1-2.

No.	Compounds	TintGOBP1		TintGOBP2	
		IC50 (μM)	Ki (μM)	IC50 (μM)	Ki (μM)
1	Linalool	16.65	16.31	23.70	22.08
2	(Z)-3-Hexen-1-ol	20.35	19.93	18.68	17.42
3	Acetophenone	24.57	24.07	-	-
4	Limonene	20.47	20.04	14.23	13.31
5	Methyl salicylate	22.67	22.20	-	-
6	Terpineol	24.36	23.86	17.88	16.70
7	β-Ionone	10.49	10.28	18.79	17.52
8	Tetradecane	-	-	-	-
9	Pentadecane	-	-	-	-
10	Hexadecane	-	-	-	-
11	Heptadecane	-	-	-	-
12	Octadecane	-	-	-	-
13	Nonadecane	-	-	-	-
14	Eicosane	-	-	-	-
15	Heneicosane	26.34	24.84	-	-
16	Cedrol	-	-	-	-
17	Nonanal	-	-	-	-
18	Decanal	-	-	-	-
19	Undecanal	-	-	-	-
20	Tetradecanoic Acid	-	-	-	-
21	Nonanoic acid	-	-	-	-
22	1-Hexadecanol	-	-	-	-

**Table 2:** The docking results of TintGOBP1-2 with different ligands.

Compounds	Cdocker Interaction Energy (Kcal/mol)		Residues Forming H-Bond with Ligand	
	GOBP1	GOBP2	GOBP1	GOBP2
Linalool	−5.33	−5.46	Thr5/Trp33	Thr5/Trp33
(Z)-3-Hexen-1-ol	−4.23	3.30	Met64/Ile90/Ile107/Ile110	Y77/Q95
Acetophenone	−3.96	-	-	-
Limonene	−5.59	−5.36		-
Methyl salicylate	−3.74	-	Met64/Glu94/Ala111	Thr5/Trp33
Terpineol	−5.64	−6.07	Thr5/Trp33	Thr5/Trp33
β-Ionone	−5.81	−6.05	-	-
Heneicosane	−6.86	-	-	-

## Data Availability

All data generated or analyzed during this study are included in this published article.

## Supplementary Materials

The following supporting information can be downloaded at https://www.mdpi.com/article/10.3390/genes17091051/s1: Table S1: *T. intacta* GOBP1-2 gene sequences derived from transcriptome data.

## References

[1] Fang N.N., Hu Y.W., Mao B., Bi J., Zheng Y., Guan C.X., Wang Y.F., Li J.H., Mao Y.K., Ai H. (2018). Molecular Characterization and Functional Differentiation of Three Pheromone-Binding Proteins from *Tryporyza intacta*. Sci. Rep..

[2] Zhao N., Li K., Ma H., Hu L., Yang Y., Liu L. (2024). Molecular Characterization of Odorant-Binding Protein Genes Associated with Host-Seeking Behavior in *Oides leucomelaena*. Int. J. Mol. Sci..

[3] Silva D., Ceballos R., Arismendi N., Dalmon A., Vargas M. (2021). Variant A of the Deformed Wings Virus Alters the Olfactory Sensitivity and the Expression of Odorant Binding Proteins on Antennas of *Apis mellifera*. Insects.

[4] Brito F.N., Moreira F.M., Melo C.A. (2016). A Look Inside Odorant-binding Proteins in Insect Chemoreception. J. Insect Physiol..

[5] Shi S. (2018). Study on Olfactory Mechanism of Host Selection on *Chilo auricilius* (Dudgeno). Master’s Thesis.

[6] Duan S.G., Mao I., Sun S.F., Chen R.D., Abdelkhalek S.T., Wang M.Q. (2025). Key Site Residues of *Cnaphalocrocis medinalis* Odorant-binding Protein 13 CmedOBP13 Involved in Interacting with Rice Plant Volatiles. Int. J. Biol. Macromol..

[7] Cheng X.J., Cai L.J., Zheng L.S., Qin J.M., Huang Y.P., You M.S. (2016). Cloning, Expression Profiling and Binding Characterization of the OBP2 Gene in the Diamondback Moth, *Plutella xylostella* (Lepidoptera: Plutellidae). Acta Entomol. Sin..

[8] Peng Z., Huang L., Zhao S.G., Lv J.H., Zhao H.T. (2021). Expression and Ligand Binding Characterization of the Odorant Binding Protein AcerOBP14 of *Apis cerana*. Acta Entomol. Sin..

[9] Rana A., Sharma D., Choudhary K., Kumari P., Ruchika K., Yangchan J., Kumar S. (2024). Insight into Insect Odorant Binding Proteins: An Alternative Approach for Pest Management. J. Nat. Pestic. Res..

[10] Zheng Z.K., Wu R.H., Li H.Y., Chen M.Z., Ting X.H., Zhao L.H., You X.T., Wu W.L., Zheng Q.K. (2023). Current Situation and Countermeasures of Using Trichogramma to Control Sugarcane Borer in Zhanjiang Sugarcane Area. Sugarcane Ind..

[11] Hu Y., Liu Y., Bi J., Chen Y., Zheng Y., Mao Y., Mao Y., Xu H., Guan C., Chen Y. (2020). Field Evaluation of Sex Pheromones and Binding Specificity of Pheromone Binding Protein 4 in *Tryporyza intacta* (Lepidoptera: Crambidae). Sci. Rep..

[12] Fu J.T., Sun D.L., Lu Y.L., Chen L.J., Zhao H.H., Dai S.X., An Y.X. (2022). Benefit Analysis of Three Types of Green and Simple Control Technology Models for Sugarcane Stem Borer. Sugarcane Ind..

[13] Zhang X.P., Zhan L., Liu Y.Y., Hu Y.W., Ai H. (2022). Research progress on the damage and control of *Proceras venosatum* and *Chilo infuscatellus* in sugarcane. Sugar Crops China.

[14] Ruhela K.S., Ruhela M. (2024). The Control of the Sugarcane Pest Top Borer (*Scirpophaga nivella* FAB*) through Many Approaches as Integrated Pest Management. J. Adv. Biol. Biotechnol..

[15] Xiong Y., Wang H., Zhou X., Wang L.L., Wang J., Wang J.H., Wu S.R. (2021). Comprehensive Prevention and Treatment Measures for Sugarcane Borer: A Review. J. South. Agric..

[16] Zheng X.H., Ma X.X., Dong B., Li C.Y., Yan Z.Y. (2021). Review of Sugarcane Borer Ecological Control Techniques. Anhui Agri. Sci. Bull..

[17] Wu F., Zheng L., Qiu Y.L., Li H.L. (2021). Research Progress of Olfactory Binding Proteins in Insects. Acta Entomol. Sin..

[18] Li M.A., Chen L.Z., Liao F., Zhao Y., Qin X.C., Wang M., Pan Q.Y., Wei L.S., Huang L.D. (2024). Sugarcane Borers: Species, Distribution, Damage and Management Option. J. Pest Sci..

[19] Alexandre M., João I.M.B., Elvira R., Dori E.N., Marat R. (2023). Dynamics and Biological Control of the Sugarcane Borer with Two Parasitoids. Ecol. Model..

[20] Liu N.Y., Yang K., Liu Y., Xu W., Anderson A., Dong S.L. (2015). Two General-odorant Binding Proteins in Spodop Tera Litura are Differentially Tuned to Sex Pheromones and Plant Odorants. Comp. Biochem. Physiol. A Mol. Integr. Physiol..

[21] Chen W.Y., Yang H.H., Gu N., Li Q.J., Zhu Y.X., Zhang N.Y. (2024). Identification of Attractants for Adult *Spodoptera Litura* Based on The Interaction Between Odorant-Binding Protein 34 and Host Volatile. Pest. Biochem. Physiol..

[22] Li J.Y.C. (2022). Studies on Binding Characteristics of Odorant-Binding Proteins and Mulberry Leaf Volatiles in *Glyphodes pyloalis* and Its Parasitoid Wasps. Master’s Thesis.

