# Supplementary material for: Functional Characteristics of General Odorant Binding Proteins in the Sugarcane Borer (Tryporyza intacta)
Source: Genes (Basel). 2026 Aug 30;17(9):1051. doi: 10.3390/genes17091051 (PMC13606456; doi:10.3390/genes17091051)
Supplement: Supplementary file 1 [file genes-17-01051-s001.zip › genes-4248176-supplementary.pdf]

Table S1. *T. intacta* GOBP1-2 gene sequences derived from transcriptome data.

Gene name	Gene sequences
TintGOBP1	ATGCGGGACGTTACTCTGGGGTTCGGCGCCGCCCTGGATCACTGCAGGGAGGAGAGCGGACTCACT<br>GAAGAGAAAATGGAAGAATTCTTCCACTTCTGGAGCGAGAACTTCAAGTTCGAGCACCGAGAGCT<br>GGGCTGCGCCCTGCACTGCATGAGCCGCTACTTCAACCTCATGACCGACACCAGCCGCATGCACCA<br>CGAGAACACGGAGCAGTTTATCAAGTCCTTCCCCAACGGCGAGCTGCTCGCGAAGAAACTGGTCAG<br>CGTGATCCATACGTGCGAGCAGCAGTTCGAACACGAAGCAGACAACTGTTGGCGCATCCTGCACAT<br>CGCTGAGTGTTTCAAGGACACCTGCAAGCGCGACGACCTCGCACCCACCATGGAGATGCTGCTCGC<br>TGAGTTCATCATGAACACCGAAGCCTAG
TintGOBP2	ATGAGCCATGTCACTGCTCATTTTGGGAAGGCCTTAGAGGAGTGTAGAGAAGAGTCCGGCCTGTCA<br>CCGGATGTCATGGAGGAGTTCAAACACTTCTGGAGCGAGGACTTCGAGGTGGTACACCGCGAGCTG<br>GGCTGCGCCCTCATCTGCATGTCCAACAAGTTCTCTCTTCTGCACGACGACACACGCATCCACCAAA<br>TCAATATGCATGACTACATCAAGAGCTTCCCTAACGGTGATGTGCTGTCAGCTAAGATGGTGGAGCT<br>GCTGCACAACTGTGAGAAGCAGTACGATGACATCACGGATGACTGCAACCGCACAGTGAAGGTGG<br>CAGCCTGCTTCAAAGTGGACGCCAAGAAGGAGGGCATCGCGCCGGAGGTGGCCATGATCGAGGCC<br>GTCATGGAACGATACTGA
